# The prevalence of obesity in children with autism: a secondary data analysis using nationally representative data from the National Survey of Children's Health

**DOI:** 10.1186/1471-2431-10-11

**Published:** 2010-02-23

**Authors:** Carol Curtin, Sarah E Anderson, Aviva Must, Linda Bandini

**Affiliations:** 1 Department of Family Medicine & Community Health, University of Massachusetts Medical School-EK Shriver Center, 200 Trapelo Road, Waltham, MA 02452, USA; 2 Division of Epidemiology, College of Public Health, The Ohio State University, B216 Starling Loving Hall, 320 West 10th Avenue, Columbus, Ohio 43210, USA; 3 Department of Public Health & Community Medicine, Tufts University School of Medicine, 136 Harrison Avenue, Boston, MA 02111, USA; 4 Department of Pediatrics, University of Massachusetts Medical School-EK Shriver Center, 200 Trapelo Road, Waltham, MA 02452, USA; 5 Department of Health Sciences, College of Health & Rehabilitation Sciences, Sargent College, Boston University, 635 Commonwealth Avenue, Boston, MA 02215, USA

## Abstract

**Background:**

The prevalence of childhood obesity has increased dramatically in the last two decades and numerous efforts to understand, intervene on, and prevent this significant threat to children's health are underway for many segments of the pediatric population. Understanding the prevalence of obesity in populations of children with developmental disorders is an important undertaking, as the factors that give rise to obesity may not be the same as for typically developing children, and because prevention and treatment efforts may need to be tailored to meet their needs and the needs of their families. The goal of the current study was to estimate the prevalence of obesity in children and adolescents with autism.

**Methods:**

This study was a secondary data analysis of cross-sectional nationally representative data collected by telephone interview of parents/guardians on 85,272 children ages 3-17 from the 2003-2004 National Survey of Children's Health (NSCH). Autism was determined by response to the question, "Has a doctor or health professional ever told you that your child has autism?" Children and adolescents were classified as obese accordingto CDC guidelines for body mass index (BMI) for age and sex.

**Results:**

The prevalence of obesity in children with autism was 30.4% compared to 23.6% of children without autism (p = .075). The unadjusted odds of obesity in children with autism was 1.42 (95% confidence interval (CI): 1.00, 2.02, p = .052) compared to children without autism.

**Conclusions:**

Based on US nationally representative data, children with autism have a prevalence of obesity at least as high as children overall. These findings suggest that additional research is warranted to understand better the factors that influence the development of obesity in this population of children.

## Background

Obesity has become a significant health concern in children in the United States, with the prevalence of childhood obesity having tripled over the last twenty years. The current prevalence of obesity among children and adolescents in the United States ages 2-19 years is 16.3% and the prevalence of overweight is 31.9%[1]. Childhood obesity is associated with an increased risk for elevated cardiovascular risk factor levels, Type 2 diabetes, orthopedic problems, sleep apnea, and menstrual irregularities[2]. Children who are overweight or obese are more likely to be obese as adults which increases their risk for chronic diseases such as diabetes, cardiovascular disease, and certain cancers[3-5].

Despite a growing literature on the problem of childhood obesity in the general population, little research has been done to examine this problem in children with developmental disabilities, including children with autism spectrum disorders (ASDs), a population of children who may be particularly vulnerable to development of obesity by virtue of the complex behavioral, physical, and psychosocial difficulties that they experience.

Autism is a developmental disorder that has an onset in childhood, usually by 3 years of age. Autism spectrum disorders (ASDs) is a commonly used term to include the diagnostic categories of autistic disorder, PDD-Not Otherwise Specified, Asperger syndrome, childhood disintegrative disorder, and Rett syndrome (although these latter two disorders are rare). The prevalence of ASDs is currently estimated to be anywhere between 1-in-150 to 1-in-91 individuals[6,7] Individuals with ASDs exhibit delayed and disordered language development, difficulties in reciprocal communication and social skills, stereotypic behaviors, a tendency towards behavioral rigidity, and sensory and behavioral difficulties.

Only a few studies have reported data on weight status of children with ASDs. Several studies have found varying prevalences of obesity in children and adolescents [8-12]. The results from these studies suggest that the prevalence of obesity in children with autism may be as high as, if not higher than, the general population of children. However, many of these studies had small sample sizes, no comparison group, and were conducted more than ten years ago.

The purpose of the present study was to use nationally representative data from the National Survey of Children's Health (NSCH) to determine the prevalence of obesity in children with autism compared to children who do not have autism.

## Methods

The National Survey of Children's Health 2003 [13] is a module of the State and Local Area Integrated Telephone Survey (SLAITS) which was jointly sponsored by the Maternal and Child Health Bureau of the Health Resources and Services Administration and the Centers for Disease Control and Prevention's (CDC) National Center for Health Statistics. The purpose of the NSCH was to obtain state and national prevalence estimates of the physical and emotional health of children ages 0-17. The population sampled included typically developing children as well as children with special health care needs, including ASDs.

Data were collected between January 2003 and July 2004 using a computer-assisted telephone interviewing (CATI) system. Sampling was accomplished by random digit-dialing a sample of households from all 50 states and the District of Columbia and identified those households that had one or more children under the age of 18. One child per household was randomly selected to be the focus of the survey. Respondents were the parent or guardian who was most knowledgeable about the child and the child's health and health care. The final sample comprised 102,353 children ages 0-17 years. The weighted response rate was 55.3%[14].

Trained interviewers asked respondents a series of questions regarding the physical, emotional, and behavioral health of the sample child. We confined our analysis to children ages 3-17 years because autism is most often diagnosed after this age. To determine the presence of autism, respondents were asked, *"Has a doctor or health professional ever told you that your child has autism?" *The unweighted sample (i.e., the number of actual respondents) of children with autism was 483 children. This sample included children who also reported a co-occurring diagnosis of one or more other health conditions including sensory deficits, behavioral or conduct problems, a developmental delay, or physical impairment. Almost all of the subjects with autism (94%) had one or more of these additional conditions, in contrast to 17% of the comparison group.

To determine weight status, respondents were asked, *"How tall is [child] now?" *and *"How much does [child] weigh now?" *Body mass index (BMI) was calculated from these respondent-reported heights and weights and was categorized as recommended by the CDC,[15] such that children with a BMI in excess of the 95th percentile for age and sex were considered obese. A large percentage of parents were unable to report either their child's height (9.9%) or weight (3.6%), and thus BMI is missing for 11.6% of the cohort (5.5% of children with autism). As a result, the sample included 454 children with autism who had data on BMI and were between 3 and 17 years old.

Sampling weights that adjust for survey non-response and unequal selection probabilities are provided in the NSCH public-use data set to support generation of population-based estimates. Sampling weights were applied in all analyses we present. The complex sample design of the NSCH was accounted for in variance estimates using SAS Survey Procedures (PROC SURVEYFREQ, PROC SURVEYLOGISTIC, SAS V9.1, SAS Institute Inc, Cary NC). Design-adjusted Chi Square tests (PROC SURVEY FREQ) were used to compare obesity prevalence between children with autism and children not so identified. Logistic regression analysis was used to estimate the prevalence odds ratio for obesity associated with autism.

The study was reviewed and approved for exemption by the University of Massachusetts Medical School Institutional Review Board.

## Results

The prevalence of autism among U.S. children and adolescents over age 3 was estimated as 0.5346%, or 1 in 189 (SE = 0.0417; 95% confidence interval (CI), 0.4462 - 0.6096). Demographic characteristics of the sample are shown in Table 1.

**Table 1:** Demographic characteristics of children with and without autism

	Children without autism	Children with autism
Gender		
Female	49.0 (0.31)	21.0 (2.68)
Male	51.0 (0.31)	79.0 (2.68)
Racial ethnic group		
Hispanic	16.9 (0.25)	10.0 (3.18)
Non-Hispanic white	61.4 (0.31)	70.4 (4.34)
Non-Hispanic black	14.6 (0.23)	15.5 (3.86)
Multi-racial	2.9 (0.10)	1.5 (0.55)
Other	4.2 (0.18)	2.6 (0.93)
Poverty-to-income ratio		
<1	17.0 (0.28)	18.1 (4.70)
1 - <2	22.7 (0.28)	21.9 (3.29)
2 - <4	33.4 (0.29)	32.9 (3.57)
>4	26.8 (0.26)	27.0 (3.05)

Percentage (standard error) tabulated. Estimates are weighted to be representative of US children.

The prevalence of obesity in children with autism was 30.4% compared to 23.6% of children without autism (p = 0.075). Children with autism were more likely to be obese than children without autism; the unadjusted odds of obesity in children with autism was 1.42 (95% CI, 1.00, 2.02, p = .052) compared to children without autism. These findings are presented in Table 2.

**Table 2:** Obesity in children with autism compared to children without autism

			Children without autism	Children with autism	P value for difference
Prevalence (standard error) for obesity*			23.6% (0.27)	30.4% (3.79)	0.075
Logistic regression model				Odds ratio (95% confidence interval) for obesity for children with autism compared to those without	
	Estimate	Standard Error	P value	Odds Ratio	95% CI
	0.35	0.18	0.052	1.42	1.00, 2.02

Obesity defined as BMI-for-age greater than or equal to the 95^th ^percentile of the CDC sex-specific BMI growth chart.

## Discussion

The results of this study suggest that children with autism are at least as likely to be obese as children who do not have autism. Based on our analysis, our best estimate indicates that children with autism are 40% more likely to be obese compared with children without autism. However, because the number of children with autism assessed was small, estimates cannot be broken out by age and sex of the child, and the confidence interval for the overall prevalence of obesity in children with autism is wide. Thus, our estimate is consistent both with children with autism having the same prevalence of obesity as other children as well as children with autism being twice as likely to be obese as other children.

Although obesity is always a result of an energy imbalance, the specific factors that contribute to excess energy intake and/or low energy expenditure among various subgroups of the general population are not yet well understood. Our analyses of the National Survey of Children's Health are descriptive and not designed to explore risk factors for obesity in children with autism. For this reason we did not adjust the estimates presented for sociodemographic or other covariates. Children with ASDs may have atypical physical activity and eating patterns that are uniquely associated with the development of obesity. For example, children with ASDs are known to have motor impairments that may adversely affect their ability to participate in sports or physical activities successfully. Such motor impairments include poor motor skills, unevenness of developmental milestone acquisition, low muscle-tone, oral-motor problems, and postural instability[16-23]. In addition, children with ASDs may experience low levels of physical activity due to their impairments in social skills which may limit participation in structured activities with peers. In fact, a recent study found that praxis/motor planning in children with autism was strongly correlated with the social, communicative, and behavioral impairments that define the disorder[24].

Children with ASDs have also been reported to have unusual eating habits, most frequently described as overly selective. A handful of small studies have documented that children with ASDs have aversions to specific textures, colors, smells, temperatures, and brand names of foods, with some preferences for soft and sweet foods [25-28]. In a larger study, Schreck et al. [29] reported that children with autism demonstrated more food selectivity than typically developing children and that the children with autism preferred energy dense foods within food groups (e.g., chicken nuggets, hotdogs, peanut butter, cake, etc.). It is possible that these eating patterns may contribute to the development of obesity in this population of children.

A strength of this study is that it is based on nationally representative data and adds to the extant literature that is primarily comprised of smaller studies. However, several limitations of the current study are noteworthy. The key measures are provided by parental report as part of a telephone interview rather than direct measurement or observation. For example, height and weight were reported by parents and not independently measured. The validity of parental report of children's height and weight has recently been shown to be at variance with direct measures, particularly in young children. A recent examination of the parent-reported height and weight for children in the current NSCH data set as well as data from the 1999-2004 National Health Interview Survey (NHIS) were compared to direct measures taken in the 1999-2004 National Health and Nutrition Examination Survey (NHANES), a nationally representative survey[30] When compared to measured data obtained from NHANES, the parent-reported data in the NSCH and NHIS over-estimated overweight among younger children and under-estimated overweight among older children. This was attributed to discrepancies in reported height among very young children. The authors conclude that these findings support previous recommendations that parent-reported data should not be used to estimate overweight prevalence among preschool and elementary school-aged children. Thus, further study of children with autism that includes measured height and weight is warranted to confirm the findings we present. Other limitations include that autism was established by an affirmative answer to a single question about whether a doctor or health professional had ever told the parent the child had autism.

## Conclusions

The present study suggests that obesity is a significant problem in children with ASDs. Children with ASDs demonstrate atypical cognitive, social, motor, and behavioral difficulties that may render them more vulnerable to the development of obesity. Research is needed to establish more firmly the prevalence of obesity in children with ASDs and to examine the factors associated with obesity in this population. Findings from such lines of inquiry would have important implications for devising appropriate prevention and intervention strategies that take these children's unique needs into account.

## Competing interests

The authors declare that they have no competing interests.

## Authors' contributions

CC conceived of the study, and CC, LB, and SEA conceived of its design. SEA acquired and performed the data analysis activities. CC, LB, SEA, and AM interpreted the data, wrote and edited the manuscript, and have approved it for submission.

## Pre-publication history

The pre-publication history for this paper can be accessed here:

http://www.biomedcentral.com/1471-2431/10/11/prepub
